# Telomere length, family history, and paternal age in schizophrenia

**DOI:** 10.1002/mgg3.71

**Published:** 2014-03-11

**Authors:** Dolores Malaspina, Roberta Dracxler, Julie Walsh-Messinger, Susan Harlap, Raymond R Goetz, David Keefe, Mary C Perrin

**Affiliations:** 1 Department of Psychiatry, Institute for Social and Psychiatric Initiatives, New York University School of MedicineNew York, New York; 2 Creedmoor Psychiatric Center, NY State Office of Mental HealthNew York, New York; 3 Department of Obstetrics and Gynecology, New York University School of MedicineNew York, New York; 4 Division of Clinical Phenomenology, New York State Psychiatric InstituteNew York, New York

**Keywords:** Genetics, psychosis, gender differences

## Abstract

Leukocyte telomere length (LTL) is longer in association with advanced paternal age, but this association has not been examined along with family history (FH) in schizophrenia. LTL was measured by PCR and compared across cases and controls as part of a study to examine the characteristics of paternal age related schizophrenia. The 53 schizophrenia cases had similar mean LTL as 20 controls, although cases were significantly older than controls and overwhelmingly smoked cigarettes. Multivariate analyses showed that a FH of schizophrenia was associated with longer LTL in both male and female cases. Later paternal age was also related to longer LTL in male cases, but with shorter LTL in female cases. Male cases with older fathers and a FH had the longest LTL. The genetic architecture associated with a familial risk for schizophrenia may include pathways that lengthen LTL. Paternal aging conferred an additional increase in LTL lengthening in male cases, but reduced LTL in female cases. The gender difference in LTL for paternal aging is consistent with the severe illness features reported for female cases with older fathers and could implicate epigenetic alterations in the paternal X chromosomal region with advanced paternal age in association with the risk for schizophrenia.

## Introduction

Schizophrenia is a heterogeneous syndrome typically beginning in late adolescence or early adulthood, characterized by declining function, avolition, deficient emotional expression, and psychotic symptoms. Poor premorbid adjustment and cognitive impairment often precede onset, but schizophrenia also presents in otherwise healthy or even high achieving young people, consistent with heterogeneity in its etiopathology.

Illness risk is increased for relatives of probands, likely through rare genetic variants of large effect and common variants with small effects, which may interact with life course exposures to determine illness risk, although most cases have no family history (FH) of the disease (Svrakic et al. 2013). Another risk factor for schizophrenia is advanced paternal age (APA), demonstrated over a decade ago (Malaspina et al. 2001) and well replicated (Hubert et al. 2011). A study in the Icelandic cohort suggested that APA was related to schizophrenia risk through *de novo* mutations that increased in association with paternal aging (Kong et al. 2012).

A large number of *de novo* mutations have now been associated with schizophrenia and older paternal age. It is plausible that each of these genes can increase the vulnerability for the disease through different pathways. However, we have demonstrated that subgroups of schizophrenia cases with older fathers have decreased variability in many measures and that they differ consistently from familial cases, including having greater hypofrontality in regional cerebral metabolism (Malaspina et al. 2004), opposite gender differences with more severe symptoms in female than male cases (Rosenfield et al. 2010), and specific patterns of cognitive deficits that differ from other cases (Lee et al. 2011). These findings suggest the possibility of a final common pathway for some of the genetic changes associated with schizophrenia risk and advancing paternal age. One recent study suggests that such genes may act in a common pathway that particularly disrupts prefrontal cortical development, as supported in a recent gene network analysis (Gulsuner et al. 2013).

The mechanism whereby an array of individually rare mutations could be amplified with paternal aging is enigmatic. Genes examined from the neuropsychiatric perspective may have prominent roles in other organ systems as well. One possibility is that mutated genes that convey schizophrenia susceptibility are also acting in the male germ line to benefit the expansion of a clone of spermatogonia over aging at the expense of other clones. This hypothesis of “selfish spermatogonia” (Goriely and Wilkie 2012) has recently been applied to schizophrenia (Goriely et al. 2013).

Another molecular finding in association with APA is longer telomere length (TL) (Aviv and Susser 2013) although this has not been examined with respect to schizophrenia. Briefly, telomeres are tandem repeats of TTAGGG at both ends of each of mammalian chromosomes that form a protective cap on the chromosome along with telomere-binding proteins. TL decreases with each cell replication in somatic cells, which lack the telomere lengthening enzyme telomerase. Shortened TL is related to more rapid cell senescence and programmed cell death (apoptosis) in association with aging and disease (Armanios 2013). Telomerase in the male germ line leads to increasing TL to some degree with each cell cycle. Another possibility is that spermatogonia with genetic variability related to longer TL, through their increased likelihood for cell division, may divide and expand more rapidly with paternal aging than other spermatogonial clones, additionally accounting of the lengthening TL in offspring that occurs over paternal age. The resultant sperm may carry genetic variation supporting longer TL.

As part of a larger NIMH Challenge Grant study to examine if “paternal age related schizophrenia” is a discrete disorder, this project examined the association of leukocyte telomere length (LTL) to APA and family history (FH) of schizophrenia, two risk factors that are associated with increased risk for the disease. Herein, we tested the hypothesis that cases without FH for schizophrenia (sporadic) and later paternal age (>33 years) would differ from familial cases and cases with younger fathers, herein testing TL.

## Method

The study was conducted at New York University and approved by the Institutional Review Board and all subjects provided written informed consent. Cases with schizophrenia or schizoaffective disorder were recruited from treatment settings and healthy controls had responded to local postings and internet recruitment sites. Inclusion criteria required cases to be on stable doses of prescribed medications. Healthy comparison subjects had no Axis I diagnoses for at least 2 years, took no psychiatric medications and had no personal or family history of psychosis. For all subjects, cigarette smoking history was categorized as never, past, or current. Diagnosis was determined by best estimate diagnostic procedures that included the Diagnostic Interview for Genetic Studies (DIGS) by reliable masters level diagnosticians and FH was assessed using the Family Interview for Genetic Study (FIGS) (Nurnberger et al. 1994).

Samples of peripheral blood were processed using density-gradient centrifugation to purify lymphocytes, and cells were frozen at −80°C until analysis. Genomic DNA was extracted with DNeasy Blood and Tissue Kit (Qiagen, Valencia, CA, USA), and DNA concentration and quality was assessed by 260/280 UV nanodrop spectrophotometery; only samples with a 260/280 ratio >1.7 were included in analysis. For LTL, DNA was extracted from lymphocytes (Laboratories, Hercules, CA, USA) by quantitative polymerase chain reaction (qPCR) (Bremner et al. 2000) with iCycler real-time PCR system and several modifications. Samples were diluted to 7 ng/μL. For the PCR reaction, each well contained: 4 μL of sterile water, 5 μL (35 ng) of genomic DNA, 0.5 μL of each primer and 10 μL of iQ SYBR Green Supermix Bio-Rad (reaction buffer with dNTPs, iTaq DNA polymerase, 6 mM MgCl2, SYBR Green I, fluorescein and stabilizers) and 7 ng/μL of DNA. The telomeric primers were Telo-F (CGG TTT GTT TGG GTT TGG GTT TGG GTT TGG GTT TGG GTT), Telo-R (GGC TTG CCT TAC CCT TAC CCT TAC CCT TAC CCT TAC CCT). The primers for the reference control gene, 36B4 single copy gene, were 36B4-F (CAG CAA GTG GGA AGG TGT AAT CC) and 36B4-R (CCC ATT CTA TCA TCA ACG GGT ACA A). The concentration of all four primers was 10 μM and PCR cycle conditions consisted of an initial denaturation step at 95°C for 10 min, followed by 40 cycles of 95°C for 15 sec and 60°C for 60 sec. The next steps were: 1 min at 95°C, 1 min at 55°C and 81 repeats of 10 sec at 55°C. The reactions were set-up in triplicate in 96-well plates, each with five DNA quantity standards (serial dilutions of a reference DNA Hela cells giving final DNA quantities of between 28 and 1.75 ng/μL per reaction), one negative control and one internal control represented by 1301 line cells. Each sample was run twice on different plates. If the difference between the mean of the three values on one plate differed by >15% from the mean of the three samples of the second plate, the sample was run a third time and the mean of the two closest measurements were used. A standard curve was made by serial dilutions of known amounts of DNA from Hela cells. The amount of each subject's DNA sample was determined relative to the single reference DNA sample by the standard curve method. The telomere signal was normalized to the signal from the single-copy gene to generate a T/S ratio indicative of relative LTLs across all chromosomes in a cell. To determine the coefficient of variation (CV), which is the standard deviation/mean × 100, we ran standard cells (HeLa cells DNA) at five different concentrations. The mean and standard deviation was computed across each plate pair and averaged to calculate the inter-assay CV. The CV was only 1.4%, showing that the variation between the assays was minimal and different runs in different plates were highly comparable.

### Data analysis

Data were entered and verified using the SIR Database Management Software (SIR 2002, SIR Pty Ltd, Terrey Hills, Australia) and IBM/SPSS Statistics 20 (Armonk, NY) was used for the analyses. Descriptive statistics and distributions of all measures were examined, whether continuous or categorical, to identify key features (e.g. non-normal distribution, outliers, skewness) that impacted inferential methods. Paternal age was considered continuously and at a cut point of 33 years in the regression analyses, based on our population based finding that the mean paternal age of schizophrenia cases was 33 years (Malaspina et al. 2001), to test if cases with APA and no family history differed from other cases LTL for cases and controls ages were examined using a diagnosis by gender (2 by 2) ANOVA. Parental ages were examined using a diagnosis by gender (2 by 2) ANCOVA with participant age as the covariate. LTL was examined with a diagnosis by gender (2 by 2) ANCOVA with age included as the covariate. Additional ANCOVA analyses of LTL were performed considering gender, paternal age, and family history of psychiatric illness. Categorical measures such as smoking status were analyzed using the Chi-squared statistic.

## Results

The schizophrenia cases were significantly older than the healthy controls, although both groups had a relatively narrow age range (case mean = 42.3 ± 10.0, range = 20–56 years versus control mean = 36.8 ± 8.7, range = 23–55 years). Subject characteristics are in Table 1. There were no significant difference in mean LTL by diagnosis or gender, nor were there significant diagnosis by gender interactions. Paternal and maternal ages did not differ by diagnosis or by gender. Male and female cases had similar ages of onset. Cigarette smoking, status defined as either “never” or “current/past,” overwhelmingly characterized cases and was infrequent in controls, without any gender differences, but smoking status, expected to shorten LTL, exhibited no significant effects in any analyses. ANCOVA comparing mean relative LTL between case and control groups by gender and covarying for age showed LTL was nonsignificantly longer for cases than controls and for males compared to females. The expected negative association between age and LTL was observed in this group of healthy controls (*r* = −0.016, *P* = 0.945,) although it was not significant.

**Table 1:** Characteristics and leukocyte telomere length in schizophrenia cases and controls by gender

	Healthy controls		Schizophrenia cases		Statistics		
			
	Males (*N* = 11)	Females (*N* = 9)	Males (*N* = 32)	Females (*N* = 21)	Diagnosis	Gender	Diag./Gender
	Mean (SD)	Mean (SD)	Mean (SD)	Mean (SD)	*F*	*F*	*F*
Age (years)	36.5 (8.5)	37.2 (9.6)	41.6 (10.3)	43.3 (9.8)	4.68*	0.22	0.03
Onset age^†^ (years)	–	–	20.4 (6.5)	21.1 (7.0)	–	*t* = 0.34 n.s.	–
Telomere length	1.92 (0.76)	1.65 (0.51)	1.98 (0.80)	1.80 (0.66)	0.30	1.30	0.05
	(*N* = 10)	(*N* = 7)	(*N* = 31)	(*N* = 20)			
Paternal age (years)	29.8 (7.7)	27.4 (4.9)	31.4 (8.7)	31.0 (11.2)	0.98	0.28	0.15
	(*N* = 11)	(*N* = 8)	(*N* = 27)	(*N* = 17)			
Maternal age (years)	29.3 (8.2)	24.1 (4.0)	27.3 (7.4)	28.4 (8.7)	0.29	0.94	2.09
	(*N* = 11)	(*N* = 6)	(*N* = 25)	(*N* = 19)			
Current or past Smokers: *N* (%)	1 (9%)	1 (17%)	21 (84%)	14 (74%)	*χ*^2^ = 23.6 ***	*χ*^2^ = 0.00	
Family history (see Table 2)			26/42	20/31			

* P < 0.05; ***P < 0.001; ^†^student's *t*-test.

Leukocyte telomere length was not significantly correlated with paternal age in the healthy control group overall (*r* = −0.366, *P* = 0.15), or separately in males (*r* = −0.341, *P* = 0.34,) or females (*r* = −0.70, *P* = 0.078) and there were no gender difference in the controls’ associations between LTL and paternal age. In the cases, however, there were significant gender differences (R to Z transformation *χ*^2^ = 4.89, df = 1, *P* = 0.027), comparing positive correlations of LTL to paternal age in male cases (*r* = 0.277, *P* = 0.13) versus the negative associations of LTL to paternal age in female cases (*r* = −0.376, *P* = 0.10). The median paternal age for our sample was 31.0 years, which is not different from the mean paternal ages by gender, as reported in Table 1.

To further interrogate these gender differences, we examined FH effects in separate gender-specific regression procedures using LTL as the dependent outcome measure to examine the effects of diagnosis, age, FH, and paternal age. In males, older paternal age still tended to be related to longer telomeres (*β* = 0.350, *P* = 0.070) without any other trend or significant predictors. In the females, by contrast, these analyses showed separate opposite effects for FH and APA, wherein FH was related to longer LTL (*β* = 0.447, *P* = 0.055), whereas APA conversely predicted shorter LTL (*β* = −0.528, *P* = 0.016). Based on our observations in this preliminary sample the effect size is small (*d* = 0.157) and power is 9%.

Finally, we tested the study hypothesis that sporadic cases with APA would differ in LTL from familial cases and from sporadic cases with younger fathers. A three group ANOVA with two categories for paternal age (cutoff score of 33 years) and for FH (positive or negative), accounting for gender, demonstrated a significant interaction between these three groups and gender (*F*_2,44_ = 4.90, *P* = 0.012). Male cases with APA had significantly longer LTL. Those with APA and a positive FH had showed significantly longer LTL than cases with younger fathers (*P* = 0.004). As APA is related to LTL in some other studies, vide supra, we conducted another post hoc analysis that included healthy control males and here again found that the male cases with older fathers and a positive family history exhibited significantly longer LTL (*P* = 0.037).

Lastly we compared if a family history of psychosis conferred a different effect on LTL in the cases with older fathers than a family history of a nonpsychotic condition. Finer separation of the cases with older fathers based on these more nuanced family history assessments (Table 2) showed longer LTL for male offspring of older fathers with a family history of psychosis and for males with a family history of a nonpsychotic psychiatric diseases than for male cases with no family psychiatric history. All male subgroups with older fathers had longer LTL than male healthy controls and male cases with younger fathers. The female cases did not show these effects for the subgroups with older fathers. ANCOVA showed this gender difference (*F* = 7.36, *P* = 0.009) and a significant group by gender interaction (*F* = 3.05, *P* = 0.036), without a group effect (*F* = 1.422, *P* = 0.238).

**Table 2:** Finer subgroup measurements of leukocyte telomere length in the subjects, by gender

Groups	Gender	Mean LTL	SD	*N*
Healthy controls	Male	1.917	0.757	11
	Female	1.650	0.505	9
Cases with paternal age ≤ 32 years	Male	1.660	0.169	16
	Female	1.887	0.905	11
Cases with paternal age > 33 years+ family history of psychosis	Male	2.694	1.581	4
	Female	1.759	0.075	3
Cases with paternal age > 33 years + family history of only nonpsychotic psychiatric illness	Male	3.004	1.273	3
	Female	1.652	0.297	5
Cases with paternal age > 33 years Without family history (only males)	Male	2.052	0.538	6

## Discussion

The study showed that advancing paternal age is associated with significantly longer LTL in males with schizophrenia and that paternal aging conversely predicted shorter LTL in female schizophrenia cases. In males and females, however, the analyses suggested that a positive family history is associated with longer LTL in males and females.

The demonstration that two risk factors for the disease, a family history and older fathers, are both linked to longer telomere lengths suggests that the molecular underpinnings of telomere length may also contribute to the genetic susceptibility for a subtype of schizophrenia, particularly in male cases. Bolstering the notion that longer LTL may occur in some portion of subjects with the schizophrenia syndrome, it was of interest that cigarette smoking and older age in the cases, which are frequently related to shorter LTL in other studies, did not predict shorter LTL in the schizophrenia group.

Longer LTL is generally associated with health and longevity, although occasionally with cancer risk (Kalmbach et al. 2013), whereas this study linked longer LTL with a FH of psychiatric illness in schizophrenia cases and with APA in male cases. Given that the mean LTL did not significantly differ between the cases and controls it seems unlikely that the longer LTL is directly producing the pathology related to the disease. However, it is plausible that longer LTL is indirectly associated with the risk for the disease through shared genetic susceptibility with schizophrenia, perhaps in a pathway that also lengthens telomeres. If so the LTL may be an important molecular marker for a subgroup of schizophrenia cases. It has been proposed that later paternal age may increase the health of the population through the lengthening of LTL that such an effect could offset risks for schizophrenia and other neurodevelopmental disorders (Aviv and Susser 2013). If replicated, the current finding that schizophrenia risk is related to the lengthening of LTL might need to be considered in balancing out the risks and benefits of APA for the benefit of the population wth respect to LTL.

While family history and APA may both have lengthened LTL in males, the two factors had opposite effects in female cases. The gender differences in which a paternal factor is related to a particular characteristic in only female offspring (herein being shorter LTL) is consistent with paternal X chromosome effects, as only the X chromosome is transmitted from fathers to daughters. A disruption of this pathway with paternal aging, attendant by decreased fidelity in the male germ line, is consistent with epigenetic dysfunction for X chromosome imprinting may be at play in the etiology of schizophrenia in female offspring with older fathers. X chromosome effects have been proposed for schizophrenia risk in females. One study found an astonishing 9-fold (95% CI 3.9–19.8) relative risk for schizophrenia recurrence in offspring of older fathers for sisters of affected female probands, compared to only a 3.3-fold (95% CI 1.6–6.6) increase for brothers of affected males with older fathers in the Jerusalem Perinatal Birth Cohort (Perrin et al. 2010). An increased risk for schizophrenia in association with older maternal grandfathers also supports the possibility of paternal age related X chromosome effects (Frans et al. 2011). The data was not sufficient to examine if maternal or paternal family histories of psychosis were differentially related to the longer LTL, although there is a report in the literature that APA is associated with a maternal history of schizophrenia, but not with a paternal history (Miller et al. 2011). As to phenotype, a hypothesis independent cluster analytic approach identified a group of females with older fathers based on specific cognitive and symptoms profiles (Lee et al. 2011), further supporting this mechanism as relevant to a subgroup of female cases.

It is interesting to compare these findings on schizophrenia to the results of an Amish population study of LTL by Njajou et al. (2007). They reported that a significant proportion of LTL was explainable by heritability of LTL and another portion was explained by paternal age. In this current study the familial effect we identified was for psychiatric illness and not for telomere length, however, again begging the question if there is overlap in the genetic substrates of LTL and the risk pathways for schizophrenia. Notably, paternal age effects were not observed in our small sample of controls. These subjects had the unusual ascertainment bias of having volunteered to be in a psychiatric research study. Another perspective about the controls is that they were a rarefied group of individuals having rigorous research interviews to exclude any psychiatric diagnosis in the last 2 years, psychiatric medications and any personal or family history of psychosis. In our cases, by contrast, the findings are consistent with the proposal that paternal age has an influence on LTL (Njajou et al. 2007) in the male cases. Their observation that daughters’ LTL was related to paternal lifespan but not with maternal lifespan also suggested an X chromosome imprinting mechanism for TL regulation. This conclusion may bolster our contention that an abnormality in the X linked epigenetic process contributing to the LTL in daughters of older fathers is related to the development of schizophrenia.

Telomere length is highly heritable across generations, and both genetic and epigenetic factors contribute to the inherited lengths, including *de novo* mutations. Further research may elaborate a role for genes in the RTK/RAS cascade, which are associated with genetic diseases and with paternal age (Goriely and Wilkie 2012; Goriely et al. 2013) with respect to telomere length. The regular linear lengthening of telomeres as men age may be associated with the increasing risk for schizophrenia (Malaspina et al. 2001). Following birth, TL shortens with age with cell divisions and environmental exposures, as telomerase is expressed in only male germ cells, stem cells, and cancer cells (Kalmbach et al. 2013). Despite the many traumas associated with schizophrenia, their excessive cigarette smoking, unhealthy lifestyles and risks for metabolic conditions, the cases in this study, who were also older, did not have reduced mean LTL lengths compared to the controls as might be expected.

As to the study goal to examine if sporadic cases with older fathers would differ in LTL compared to other cases, our results are mixed. Paternal aging did extend LTL, but only in males, and family history also extended LTL in both males and females. Notably the males with both a FH and with APA had the longest LTL, whether the family risk was through psychosis or other psychiatric disease, indicating that these are separate factors. The findings also support that our effects were not explained by failures to ascertain FH in cases with older fathers. The gender differences with respect to LTL and paternal age in the disease were not expected, but the finding shines a light on the purported X chromosome effects for the disease from other studies, as reviewed.

These results represent the entirety of the case and control samples recruited for this funded proposal to examine if paternal age related schizophrenia is a discrete disorder within the schizophrenia syndrome. Confirmatory and additional studies should use larger samples. We did not test the influence of maternal age on the LTL female offspring owing to the small number of female cases in this preliminary study, but further work should explore this association. Future studies could be nested within epidemiological cohorts. Until such time as these findings are replicated these results should be considered to be preliminary. Countering the small numbers is the rich clinical depth of this study and state of the art diagnostic procedures and that the study was specifically designed to examine if paternal age related schizophrenia was a discrete disorder within the schizophrenia spectrum. Finally, the qPCR methodology for estimating LTL is well-established, but the samples were of leukocytes and not of neurons. It should be noted that LTL is highly correlated with TL of other tissues and these elongated-telomere cases are likely to have long TL in other tissues. However, we still cannot exclude significantly shorter TL in neural stem cells (Aviv and Susser 2013).

These results should encourage research concerning the risk pathways that could associate telomere length with schizophrenia and explore if LTL is a useful biomarker for etiology or treatment development in the disease.
